# Overexpression of *β*2-microglobulin is associated with poor survival in patients with oral cavity squamous cell carcinoma and contributes to oral cancer cell migration and invasion

**DOI:** 10.1038/sj.bjc.6604698

**Published:** 2008-10-07

**Authors:** C-H Chen, C-Y Su, C-Y Chien, C-C Huang, H-C Chuang, F-M Fang, H-Y Huang, C-M Chen, S-J Chiou

**Affiliations:** 1 Department of Otolaryngology, Chang Gung Memorial Hospital-Kaohsiung Medical Center, Chang Gung University College of Medicine, Kaohsiung, Taiwan; 2 Kaohsiung Chang Gung Head and Neck Oncology Group, Chang Gung Memorial Hospital-Kaohsiung Medical Center, Kaohsiung, Taiwan; 3 Department of Pathology, Chang Gung Memorial Hospital-Kaohsiung Medical Center, Chang Gung University College of Medicine, Kaohsiung, Taiwan; 4 Department of Radiation Oncology, Chang Gung Memorial Hospital-Kaohsiung Medical Center, Chang Gung University College of Medicine, Kaohsiung, Taiwan; 5 Department of Biochemistry, Faculty of Medicine, Kaohsiung Medical University, Kaohsiung, Taiwan

**Keywords:** *β*2-microglobulin, immunohistochemistry, invasion, migration, oral cavity squamous cell carcinoma

## Abstract

*β*2-Microglobulin (*β*2M), a component of MHC class I molecules, is believed to be associated with tumour status in various cancers. In this study, we examined the expression of *β*2M at different malignant stages of oral cavity squamous cell carcinoma (OCSCC). To determine the possible correlation between *β*2M expression and various clinical characteristics, 256 samples from patients with OCSCC were evaluated by immunohistochemical staining. Strong *β*2M expression was significantly correlated with a relatively advanced tumour stage (*P*<0.001), positive nodal status (*P*<0.001), and TNM stage (*P*<0.001). The cumulative 5-year survival rate was significantly correlated with a relatively advanced tumour stage (*P*<0.001), positive nodal status (*P*<0.001), TNM stage (*P*<0.001), and *strong* expression of *β*2M (*P*<0.001). Thus, elevated *β*2M expression is an indicator of poor survival (*P*<0.001). In addition, we extended our analysis of *β*2M expression to the FaDu and SCC25 oral cancer cell lines. *β*2-Microglobulin expression was positively correlated with cell migration and invasion in *β*2M-overexpressing transfectants in Transwell chambers. The suppression of *β*2M expression using small interfering RNA (siRNA) was sufficient to decrease cell migration and invasion *in vitro*. Taken together, our results suggest that *β*2M expression in the tissues is associated with survival and may be involved in tumour progression and metastasis in OCSCC.

*β*2-Microglobulin (*β*2M), an 11.7-kDa polypeptide expressed on the surface of almost all cells in the body, forms complexes with major histocompatibility complex (MHC) class I molecules, which are believed to function in antigen presentation to cytotoxic (CD8+) T lymphocytes (Margalit *et al*, 2006). *β*2-Microglobulin is present as a soluble protein at low levels in the serum, urine, and other bodily fluids under physiological conditions; however, its level is elevated in patients with kidney failure and certain malignancies, including solid and liquid tumours (Klein *et al*, 1996; Tsimberidou *et al*, 2008). The increased tissue/serum level of *β*2M is associated with a high tumour burden and poor prognosis. Thus, the level of *β*2M has become one of the most important prognostic factors and predictors of survival in patients with certain cancers (Lee *et al*, 2000; Madjd *et al*, 2005; Tsimberidou *et al*, 2008). Numerous reports, however, have indicated that a rise in the level of *β*2M does not necessarily indicate a poor prognosis, suggesting that the changes in *β*2M expression differ between premalignant and malignant tumours (Mahrle *et al*, 1982; Korkolopoulou *et al*, 1996; Feenstra *et al*, 2000; Palmisano *et al*, 2001). The first studies of cancer cells with upregulated or downregulated *β*2M expression were initiated two decades ago; however, to date, few articles have shown that a decrease in the cell surface concentration of *β*2M is associated with a poor prognosis in malignant cases of oral cavity squamous cell carcinoma (OCSCC) (Prime *et al*, 1987; Feinmesser *et al*, 2004). Instead, prominent staining for *β*2M in tumours is associated with an improved clinical outcome. Oral cavity squamous cell carcinoma is currently the most frequently detected head and neck cancer in Southeast Asia. In Taiwan, OCSCC is the fifth most common malignancy in men, although it is highly curable at an early stage (Lu *et al*, 2007). The identification of biomarkers for evaluating the progression of OCSCC is therefore urgent. The aim of this study was to investigate the clinicopathological significance of *β*2M expression according to tumour status in patients with OCSCC. Using *β*2M-overexpressing transfectants, we found a statistically significant correlation between elevated *β*2M expression and oral cancer cell invasion and migration.

## Materials and methods

### Patients and tumour samples

The subjects included 256 patients without previous radiotherapy and/or chemotherapy who underwent primary surgical resection between October 1996 and August 2005 for the treatment of OCSCC. Clinicopathological information, including sex, age, primary tumour stage (T), nodal status (N), and tumor-node-metastasis (TNM) stage, was obtained from each patient's clinical records and pathologic reports. Tumor-node-metastasis status was assigned according to the 1997 American Joint Committee on Cancer staging system. Tumor-node-metastasis was defined as size or direct extent of the primary tumour (T, 1–4) that spreads to regional lymph nodes (N, 0–3) and develops distant metastasis (M, 0/1). This study was approved by the Medical Ethics and Human Clinical Trial Committee at Chang Gung Memorial Hospital, Taiwan. The study subjects included 17 women and 239 men with an average age of 50.9 years (range: 26–87). Thirty-nine of the patients were classified as T1, 55 as T2, 64 as T3, and 98 as T4. One hundred and fifty-three patients were classified as N0, 38 as N1, 48 as N2b, 13 as N2c, and 4 as N3. Thirty-four patients were classified as TNM stage I, 38 as stage II, 61 as stage III, and 123 as stage IV. The mean follow-up period was 49.3 months (range: 2–141).

### Antibodies and reagents

Polyclonal antibodies against human *β*2M and HLA class I were purchased from Santa Cruz Biotechnology (Santa Cruz, CA, USA). Monoclonal antibodies against *β*-actin were obtained from Sigma (St Louis, MO, USA). Anti-HA antibodies were purchased from Roche Biochemicals (3F10; Indianapolis, IN, USA). Horseradish peroxidase (HRP)-conjugated anti-rabbit IgG, HRP-conjugated anti-mouse IgG, FITC-conjugated anti-rabbit IgG, and TRITC-conjugated anti-mouse IgG were purchased from Jackson ImmunoResearch laboratories (Bio/Can Scientific, Mississauga, ON, Canada). An HRP/Fab polymer conjugate kit and DAB were obtained from Zymed (PicTure™-Plus Kit; South San Francisco, CA, USA). SuperScript II Reverse Transcriptase and all PCR and cell transfection reagents were purchased from Invitrogen (Carlsbad, CA, USA). All cell culture-related reagents were purchased from Gibco-BRL (Grand Island, NY, USA).

### RNA extraction and semiquantitative reverse transcription-PCR

Tissue samples were frozen in liquid nitrogen and stored at −80°C before RNA extraction. The tissues were homogenised using a Mixer Mill Homogenizer (Qiagen, Crawley, West Sussex, UK). Total RNA was extracted from the tissue samples using an RNeasy Mini Kit (Qiagen) according to the manufacturer's instructions. The total RNA (2 *μ*g) was reverse-transcribed into cDNA using SuperScript II Reverse Transcriptase. Polymerase chain reaction was performed using 1 *μ*l of the reverse transcription product in a reaction volume of 25 *μ*l. The primers used were *β2M* forward: 5′-CTCACGTCATCCAGCAGAGA-3′ and reverse: 5′-CGGCAGGCATACTCATCTTT-3′; and *GAPDH* forward: 5′-GAAGGTGAAGGTCGGAGTC-3′ and reverse: 5′-GAAGATGGTGATGGGATTTC-3′. *GAPDH* was used as an internal control to normalise the relative amount of cDNA in each reaction. The number of cycles corresponding to the logarithmic phase of amplification for *β2M* was determined before the start of our experiments. The PCR mixture contained 10 mM Tris-HCl (pH 8.3), 1.5 mM MgCl_2_, 50 mM KCl, 200 mM dNTPs, 2 mM each primer, and 1 U of Ex Taq Polymerase (Takara, Tokyo, Japan). The programme included 25 cycles at 94°C for 1 min, 56°C for 1 min, and 72°C for 2 min. The reverse transcription-PCR (RT–PCR) products were separated on 2% agarose gels and stained with 0.5 *μ*g ml^−1^ ethidium bromide. The *β2M* product was 213 bp long.

### Immunoblot analysis

Immunoblotting was carried out according to standard procedures. For tissue protein extraction, samples were frozen and homogenised in lysis RIPA (radioimmunoprecipitation assay) buffer (50 mM Tris-HCl (pH 7.5), 150 mM NaCl, 1% NP-40, 0.5% Na-deoxycholate, and 0.1% SDS). The protein concentration in each sample was estimated by Bio-Rad protein assay (Hercules, CA, USA). Equal amounts of protein (50 *μ*g) were electrophoresed on reducing 10% SDS–polyacrylamide gels and then transferred to polyvinylidene difluoride membranes. After blocking with TBS/5% bovine serum albumin (BSA), antibodies against *β2M*, HA, and *β*-actin were incubated with the membranes at room temperature for 1 h. The resulting IgGs were detected using HRP-conjugated secondary antibodies and developed using Western Lighting reagent.

### Immunohistochemistry

Normal oral mucosa and adjacent non-tumour and tumour OCSCC tissue samples were selected by a pathologist on the basis of diagnosis and microscopic morphology. Normal oral mucosa and tumour tissues were fixed with 10% buffered formalin embedded in paraffin and decalcified in 10% EDTA solution. Representative blocks of the formalin-fixed, paraffin-embedded tissues were cut to 4 mm thickness and deparaffinised with xylene and rehydrated in a series of ethanol washes (100, 90, 80, and 70%). Slides were washed with phosphate-buffered saline (PBS) and treated with 3% H_2_O_2_ for 30 min to block endogenous peroxidase activity. Next, the sections were microwaved in 10 mM citrate buffer, pH 6.0, to unmask the epitopes. After antigen retrieval, the sections were incubated with diluted anti-*β*2M (1 : 200) or anti-HLA-I antibody (1 : 200) for 1 h followed by PBS wash. Horseradish peroxidase/Fab polymer conjugate (PicTure™-Plus kit; Zymed) was then applied to the sections for 30 min followed by PBS wash. Finally, the sections were incubated with peroxidase substrate diaminobenzidine for 5 min to develop the signals. A negative control was run simultaneously by omitting the primary antibody. To grade for the intensity of *β*2M immunostaining, the level of immunoreactivity in the immunostained tissues was evaluated independently by two pathologists who were blinded to the subjects' clinical information. To evaluate the expression of *β*2M, the tissue sections were examined under a microscope at a magnification of × 200. The intensity of staining was classified according to a four-level scale: −, no or faint staining in a few tumour cells; +, weak cytoplasmic staining in most tumour cells; ++, diffuse cytoplasmic staining in groups of tumour cells; and +++, diffuse cytoplasmic staining in most of the tumour cells (Yagasaki *et al*, 2003). Levels − and + were defined as weak *β*2M expression, whereas levels ++ and +++ were defined as strong expression. A sample was considered positive if 1–5% of the tumour cells showed positive staining as described (Mehta *et al*, 2008). Those samples in which positive staining were detected in over 50% of the tumour cells were considered strongly positive for *β*2M.

### Construction of the human *β*2M plasmid

Two primers, 5′-atgtctcgctccgtggcctt-3′ and 5′-TTACATGTCTCGATCCCACT-3′, were used to amplify the *β2M* cDNA from the SCC25, a cell line derived from OCSCC cDNA library. SCC25 cells were homogenised using a Mixer Mill Homogenizer (Qiagen). Total RNA from SCC25 cells was extracted using the RNeasy Mini kit (Qiagen) according to the manufacturer's instructions. Total RNA (2 *μ*g) was reverse-transcribed into cDNA by SuperScript II reverse Transcriptase. The PCR mixture contained 10 mM Tris-HCl (pH 8.3), 1.5 mM MgCl_2_, 50 mM KCl, 200 mM dNTP, and 2 mM of each primer with 1 U of Ex Taq Polymerase (Takara). The PCR involved denaturation at 94°C for 1 min and reaction at 62°C for 1 min and 72°C for 2 min for a total of 35 cycles. The purified PCR product was constructed and sequenced. GenBank accession number for *β2M* is NM_004048. The full-length *β*2M was subcloned into the pcDNA3.1, a HA-tagged expression vector.

### Cell culture establishment of stable clones, and transient transfection of *β2M* siRNA

FaDu and SCC25 cells were grown in Dulbecco's modified Eagle's medium (DMEM) containing 10% heat-inactivated foetal bovine serum (FBS) and 100 U ml^−1^ penicillin and streptomycin. Transient transfection of FaDu and SCC25 cells with HA-tagged *β2M* was achieved using Lipofectamine according to the manufacturer's instructions. FaDu and SCC25 cells stably expressing *β*2M were selected using 400 *μ*g ml^−1^ G418 (Calbiochem Novabiochem, San Diego, CA, USA). Each clone was harvested and analysed for exogenous *β*2M expression by western blotting. Each stable clone was then lysed in extraction buffer (20 mM piperazine-*N*,*N*′-bisethane sulphonic acid, pH 7.2, 100 mM NaCl, 1 mM EDTA, 0.1% 3-[(3-cholamido propyl)-dimethylammonio]-2-hydroxy-1-propanesulphonic acid, 10% sucrose, 1 mM DTT, 1 mM PMSF, and 1 mM Na_3_VO_4_) as described earlier (Hsu *et al*, 2004). Double-stranded synthetic RNA oligomers (Ambion, Austin, TX, USA) (5′-UUGCUAUGUGUCUGGGUUUtt-3′ and 5′-AAACCCAGACACAUAGCAAtt-3′), deduced from human *β2M*, and one negative control siRNA (5′-uucaugugucugugguguutt-3′ and 5′-AACACCACAGACACAUGAAtt-3′) were used in our siRNA experiments (Nomura *et al*, 2006).

### Flow cytometry

For analysis of the expression level of *β*2M and HLA-I molecule in cancer cell surface, we used flow cytometry to perform the experiments. FaDu cells were trypsinised and harvested in PBS. The cells were incubated with a 1 : 100 dilution of anti-*β*2M and anti-HLA class I (Santa Cruz Biotechnology) antibodies or an equivalent concentration of isotype-specific mouse IgG in PBS with 1% BSA overnight at 4°C with gentle agitation. After washing, cells were incubated for 30 min at room temperature with 1 : 500 dilution of FITC-conjugated anti-rabbit and TRITC-conjugated anti-mouse IgG secondary antibody, then washed with PBS-1% BSA. The cell-associated fluorescence of 10 000 events per sample was analysed in a FACScan flow cytometer (Becton Dickinson, San Jose, CA, USA) using the Cell Quest software.

### Migration and invasion assay

Migration and invasion assays using FaDu/vehicle, FaDu/*β*2M, SCC25/vehicle, and SCC25/*β*2M stable clones were conducted using 24-well Transwell (8-*μ*m pore size polycarbonate membrane; CoStar, Bethesda, MD, USA) chambers. For the migration assays, 5 × 10^3^ cells suspended in 400 *μ*l of DMEM containing 10% FBS were seeded onto the upper chamber, whereas 600 *μ*l of DMEM containing 10% FBS was added to the outside of the chamber. After 24 h of culture at 37°C under 5% CO_2_/95% air, the cells on the upper surface of the membrane were removed using a cotton tip applicator, whereas the migratory cells on the lower membrane surface were fixed with methanol and stained with Giemsa (Sigma). Migration was assessed by counting the number of cells that had migrated on three independent membranes under a phase contrast microscope ( × 200); this value was then normalised against that for the vehicle cells to produce the relative ratio. For the invasion assays, 117 *μ*g of Matrigel (BD Biosciences, San Jose, CA, USA) was added to the upper surface of the membrane and allowed to gel overnight at 37°C. Matrigel is a commercial product extracted from a mouse sarcoma rich in extracellular matrix proteins. The major component is laminin, followed by collagen IV and heparin sulphate proteoglycans. In total, 1 × 10^4^ cells in 400 *μ*l of DMEM containing 10% FBS were seeded onto the upper chamber, whereas 600 *μ*l of DMEM containing 10% FBS was added to the outside of the chamber. The subsequent steps were the same as in the migration assays.

### Statistical analysis

Several clinicopathological factors were evaluated, including sex, age (⩽59 years versus ⩾60 years), T stage (T1, T2 *vs* T3, T4), N status, and TNM stage (stage I, II *vs* stage III, IV). Fisher's exact test was used to evaluate the correlation between the clinicopathological variables and the *β*2M expression level. A *P*-value<0.05 was considered to be significant. The clinicopathological variables and *β*2M expression data were taken into account for the analysis of survival based on the Kaplan–Meier method; statistical significance was defined as a *P*-value<0.05 as assessed by the log-rank test. To determine the effect of specific prognostic factors on survival, a multivariate analysis was performed according to Cox's regression model.

## Results

### Analysis of *β*2M Overexpression in OCSCC

**Figure 1:**
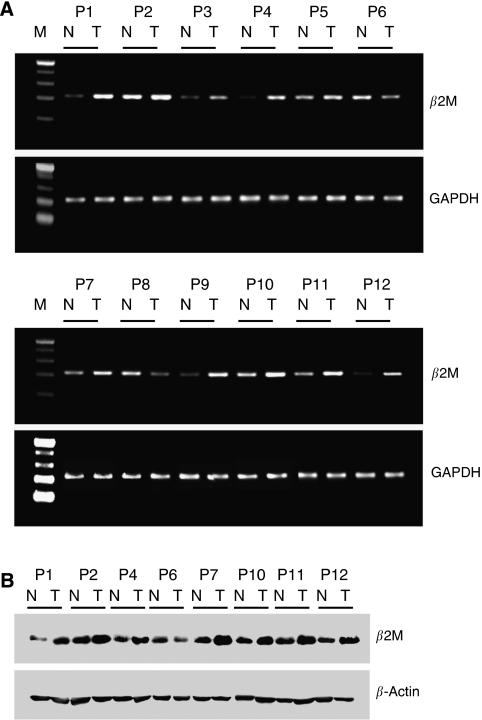
*β*2-Microglobulin (*β*2M) is overexpressed in OCSCC. (**A**) RT–PCR analysis of *β2M* expression in OCSCC samples (T) *vs* that in adjacent non-tumour tissues (N). *GAPDH* was used as an internal loading control to normalise the amount of RNA. (**B**) Western blot analysis of *β*2M expression in eight paired patients with oral cancer. Total protein extracts were prepared from adjacent non-tumour (N) and tumour (T) tissues and probed with polyclonal antibodies against human *β*2M. *β*-Actin was used as a control for equal protein loading.

The mRNA expression of *β2M* was evaluated by semiquantitative RT–PCR using a panel of paired tumour/adjacent non-tumour tissue samples. Compared with the adjacent non-tumour tissues, almost all of the OCSCC samples displayed elevated *β2M* expression; only two of them showed downregulation of *β2M* (Figure 1A). Similar results were obtained for the protein expression of *β*2M (Figure 1B). The band corresponding to *β*2M was detected in each of the tumour samples. The identity of the band was confirmed by preincubating the sample extracts with anti-*β*2M antibodies followed by immunoprecipitation (data not shown). A high percentage of the OCSCC samples (seven of eight, 87.5%) showed enhanced *β*2M expression as compared with the adjacent non-tumour tissues. Taken together, these data indicate the elevated expression of *β*2M in OCSCC.

### Association of *β*2M expression with various clinicopathological features

**Figure 2:**
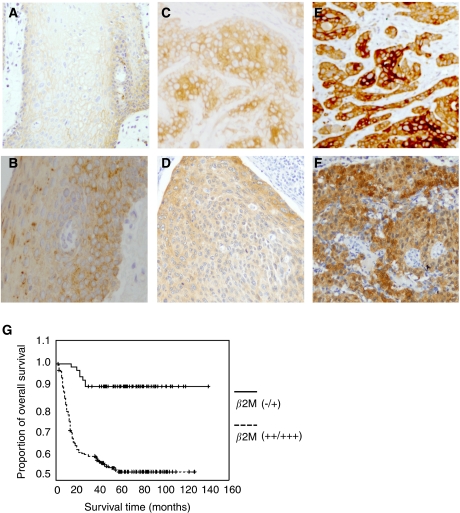
Immunohistochemical staining for *β*2M and overall survival in patients with OCSCC. (**A**–**F**) The *β*2M intensity on tissues was evaluated by immunohistochemical staining. (**A**) The *β*2M protein was localised at plasma membrane in normal mucosa tissue. Tumour tissues with plasma membrane (**C**, **E**) or cytoplasmic (**D**, **F**) *β*2M staining were classified according to a two-grade scale: absent or weak staining (−/+ **C**, **D**), and strong staining (++/+++, **E**, **F**) as compared with weak *β*2M staining in adjacent non-tumour oral tissue (**B**). (**G**) The survival period for those patients (*n*=184) with strong (++/+++) tumour expression (dashed line) was significantly shorter than that for those (*n*=72) with absent or weak (–/+) expression (solid line). The difference in survival was statistically significant (*P*<0.001) according to the log-rank test. Overall survival was calculated from the time of surgery to the date of death, the event of interest, or the date of last follow-up. All statistical analyses were performed using SPSS software.

*β*2-Microglobulin expression in the cytoplasm and cytoplasma membrane of tumour epithelial cells collected at various stages (T1, T2 and T3, T4) was compared with that in normal oral mucosa and cells from the adjacent non-tumour tissues by immunohistochemistry (Figure 2A–F). The normal oral mucosa was no or very weak intensity for *β*2M staining mainly in plasma membrane (Figure 2A). Prominent staining was observed in the tumour samples (Figure 2C–F) compared with that in the adjacent non-tumour tissues, which showed no or very weak *β*2M expression (Figure 2B). It can be noted that *β*2M was found largely localised in the cytoplasm of both tumour samples (Figure 2D and F) and the adjacent non-tumour tissues (Figure 2B). In some cases, *β*2M was also expressed focally in the plasma membrane (Figure 2C and E). Our immunohistochemical data and the correlation with various clinicopathological variables are summarised in Table 1. Of the 94 patients categorised as T1, T2, 73.4% (*n*=69) of the tumours showed no or weak staining (−/+), whereas only 26.6% (*n*=25) exhibited strong staining (++/+++). In contrast, of 162 patients classified as T3, T4, 1.97% (*n*=3) showed a reduction in *β*2M staining (−/+), whereas 97.5% (*n*=159) presented with strong (++/+++) staining. Similarly, among the 103 patients who were N(+), 95.2% (*n*=98) of the tumours showed strong (++/+++) *β*2M expression, whereas only 4.8% (*n*=5) showed weak (−/+) expression. In terms of TNM stage, 100% (*n*=184) showed strong staining in the more malignant stages (III, IV), whereas 100% (*n*=72) presented with weak staining at less malignant stages (I, II). These data suggest that increased expression of *β*2M is significantly correlated with a relatively advanced tumour stage (T3, T4 *vs* T1, T2, *P*<0.001), positive nodal status (N(+) *vs* N(–), *P*<0.001), and TNM stage (III, IV *vs* I, II, *P*<0.001). In contrast, no correlation was observed between *β*2M expression and sex or age.

### Analysis of survival according to *β*2M expression

The Kaplan–Meier analysis of our immunohistochemical results for the patients with OCSCC revealed that the cumulative 5-year overall survival rate was significantly correlated with the clinicopathological characteristics and expression of *β*2M (Table 2 and Figure 2G). The disease-free survival rates for those patients in stages T1 and T2 with a negative nodal status and stages I and II were significantly higher than for those in stages T3 and T4 with a positive nodal status and stages III and IV (all *P*<0.001, log-rank test). Taken together, the overall survival rates for those patients with weak *β*2M expression (92.4%) were significantly higher than for those with *β*2M overexpression (51.4%, *P*<0.001, log-rank test). These data suggest an increased survival period for those patients with OCSCC having decreased *β*2M expression.

### Multivariate analysis

To determine whether *β*2M expression is an independent predictor of survival, Cox's regression analysis was carried out using tumour stage, lymph nodal status, TNM stage, and *β*2M expression as parameters. Our data indicated that tumour stage (hazard ratio (HR): 3.293; 95% CI: 1.877–5.778; *P*<0.001) and nodal stage (HR: 2.990; 95% CI: 1.937–4.616; *P*<0.001) were each independent prognostic factors for OCSCC (Table 3). *β*2-Microglobulin status (*P*=0.192) and TNM stage, however, were not independent predictors. Although *β*2M was not a key independent factor in our multivariate analysis, we suggested that *β*2M may play an important role in the tumour development and nodal metastatic processes in OCSCC patients according to the results of Tables 1 and 2.

### Effect of *β*2M on oral cancer cell invasion and migration

**Figure 3:**
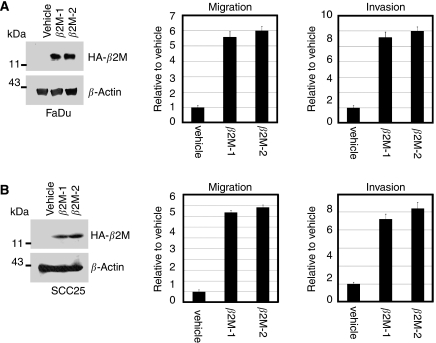
*β*2-Microglobulin (*β*2M) overexpression promotes oral cancer cell migration and invasion. (**A** and **B**, left panel) HA-tagged *β*2M stable clones of FaDu and SCC25 cells were established. Cell lysates (50 *μ*g) were then prepared and subjected to immunoblot analysis with anti-HA antibodies. (**A** and **B**, middle panel) For the migration assays, 5 × 10^3^ cells of the FaDu/vehicle, FaDu/*β*2M-1 and *β*2M-2, SCC25/vehicle, and SCC25/*β*2M-1 and *β*2M-2 stable clones were seeded onto the top of a Transwell insert. After 24 h, the cells on the topside were scraped, and the cells that had migrated to the bottom were fixed and stained with Giemsa. The relative-fold migration of the FaDu/vehicle, FaDu/*β*2M-1 and *β*2M-2, SCC25/vehicle, and SCC25/*β*2M-1 and *β*2M-2 cells was normalised against the vehicle control and are presented diagrammatically. (**A** and **B**, right panel) For the invasion assays, 1 × 10^4^ cells were seeded after the addition of Matrigel. The relative-fold invasion of the stable clones was normalised against the vehicle cells and is shown diagrammatically. The data represent the mean±s.d. of three independent experiments.

**Figure 4:**
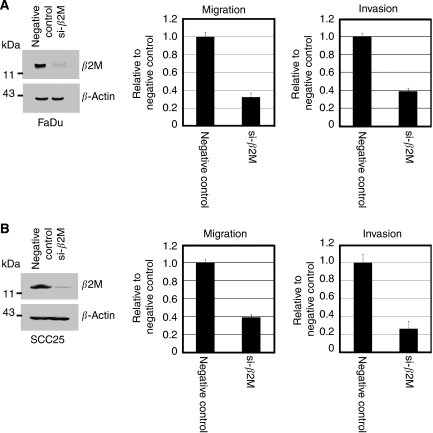
The migratory and invasive capacity of oral cancer cells is inhibited by *β*2M-mediated siRNA. (**A** and **B**, left panel) A negative control siRNA or si-*β*2M was transfected into FaDu and SCC25 cells. After 24 h, western blotting was performed using anti-*β*2M and anti-*β*-actin antibodies. (**A** and **B**, middle and right panels) The relative-fold migration and invasion values for the FaDu/si-*β*2M and SCC25/si-*β*2M cells were normalised against the negative control cells and are presented diagrammatically. The results represent the mean±s.d. of three independent experiments.

**Figure 5:**
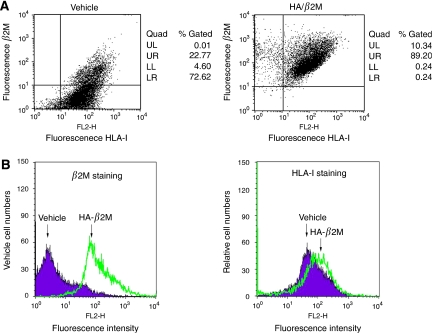
Flow cytometric analysis of the expression profile of *β*2M and HLA-I on the surface membrane of oral cancer cells. (**A**) FaDu/vehicle and FaDu/*β*2M-1 cells were co-incubated with *β*2M (2 *μ*g ml^−1^) and HLA-I (2 *μ*g ml^−1^) antibodies, as indicated. At least 10^6^ cells were analysed on a FACSCalibur flow cytometer under standard flow for 120 s. Percentage of cell gated in each quadrant. Representative results of three independent experiments. (**B**) Representative histograms on the basis of flow cytometric analysis of detached FaDu/vehicle and FaDu/*β*2M-1 cells after immunolabelling with *β*2M and HLA-I antibodies. Representative results of three independent experiments.

To determine whether *β*2M expression is correlated with cellular migration and invasion *in vitro*, two oral cancer cell lines, FaDu and SCC25, were stably transfected with an expression vector carrying human *β2M* cDNA or vector alone as a control. For each cell line, two clones were selected (FaDu/*β*2M-1, *β*2M-2 and SCC25/*β*2M-1, *β*2M-2). The level of overexpression in the FaDu/*β*2M-1, *β*2M-2 and SCC25/*β*2M-1, *β*2M-2 cells was evaluated by western blot analysis as shown in Figure 3A and B, left panels, respectively. No significant difference in proliferation rate over 24 h was identified between those cells carrying the vehicle control and those cells expressing *β*2M by MTT assay (data not shown). In addition, the morphology of the cells in each group did not change. The ability of the *β*2M-expressing transfectants to migrate across the surface of a chamber was also analysed 24 h after seeding. Compared with the vehicle-transfected FaDu cells, the migratory ability of the FaDu/*β*2M-1, *β*2M-2 cells significantly increased by 5.5- and 6.0-fold, respectively (*P*<0.001, Figure 3A, middle panel). Owing to the slightly lower level of *β*2M expression in SCC25 cells, the SCC25/*β*2M-1, *β*2M-2 cells displayed 4.6- and 5.2-fold greater migration, respectively (*P*<0.001, Figure 3B, middle panel). This indicates that a rise in *β*2M expression is positively correlated with the migration of FaDu and SCC25 cells. To clarify the role of *β*2M in invasiveness, the ability of the transfectants to pass through a Matrigel barrier was assayed. Compared with the vehicle-transfected cells, the FaDu/*β*2M-1, *β*2M-2 cells showed an 8.2- and 8.8-fold greater capacity for invasion, respectively (*P*<0.001, Figure 3A, right panel). Similarly, invasion by the SCC25/*β*2M-1, *β*2M-2 increased by 7.8- and 8.0-fold, respectively (*P*<0.001, Figure 3B, right panel). Taken together, these results suggest that an increase in the level of *β*2M increases the *in vitro* migratory and invasive capacity of FuDu and SCC25 cells. We next analysed whether the inhibition of *β*2M expression would decrease the migratory and invasive capacity of FaDu and SCC25 cells. FaDu and SCC25 cells were transiently transfected with either si-*β*2M or a negative control and two clones were selected (FaDu/si-*β*2M and SCC25/si-*β*2M). After 24 h, the cells were harvested for western blot analysis and seeded into a Transwell apparatus for migration and invasion assays. As shown in Figure 4A and B, left panels, respectively, endogenous *β*2M expression was effectively inhibited in the FaDu and SCC25 cells. The migratory ability of the FaDu/si-*β*2M and SCC25/si-*β*2M cells decreased by 70 and 64% compared to the negative controls, respectively (*P*<0.001, Figure 4A and B, middle panels). The *in vitro* invasiveness of the two transfectants decreased by 62 and 75% compared with the negative controls, respectively (*P*<0.001, Figure 4A and B, right panels). These results indicate that si-*β*2M negatively affected both the migration and invasion of the FaDu and SCC25 cells. To address the mechanistic basis for the activity of *β*2M, the expression of membranal *β*2M in the transfectants was examined by flow cytometric analysis. The representative data showed that ectopic expression of *β*2M increased cell number by 3.9-fold in the transfectants, as compared with that in the vehicle (Figure 5A). We went on to obtain a quantitative analysis of the effect of *β*2M expression on the level of HLA-1 in the transfectants. Figure 5B showed that the expression of *β*2M did not significantly elevate the level of endogenous HLA-I in the transfectants. The immunohistochemical data further confirmed that staining intensity for HLA-I had no significantly different between the adjacent-non tumour and tumour tissues in *β*2M-overexpressing specimens (Supplementay Figure 1).

## Discussion

Increased levels of *β*2M have been reported in solid and liquid malignancies, and this has allowed us to investigate the role of *β*2M beyond antigen presentation. This is the first study to explore whether the level of *β*2M expression is an important prognostic factor in OCSCC. Increased *β*2M expression was significantly correlated with tumour stage, lymph node metastasis, and survival (Tables 1 and 2). Our results strongly suggest that the level *β*2M is a risk factor for tumour progression in OCSCC (Table 2). In contrast to our results, reduced levels of *β*2M expression have been reported in cases of head and neck squamous cell carcinoma (HNSCC) and in malignancies of the oral mucosa, as the downregulation of HLA expression is frequently observed in malignancies (Prime *et al*, 1987; Koene *et al*, 2004). A similar report indicated that immunotherapy significantly restored *β*2M expression and was associated with an improved outcome in patients with HNSCC (Feinmesser *et al*, 2004). The experimental group in that study was probably too small to cover all of the tumour stages. Moreover, immunohistochemical analysis allows for the selection of small tumour fields, but it is not representative of the overall tumour, which may contain heterogeneous cells. Although tumours frequently produce a variety of ‘loss’ phenotypes (Aptsiauri *et al*, 2007), including defects in or the genomic loss of the *β*2M locus, clinical conclusions must be drawn with caution. To date, the mechanism responsible for an increase in *β*2M expression during the progression of cancer is unclear. One interpretation is that the level increases as a consequence of increased cell turnover in the tumour and an enhanced immune response to the malignant process. Another possibility is that the tumours contain three *β2M* alleles instead of one. Recently, Nomura *et al* (2006) demonstrated that *β*2M promoted growth in human renal cell carcinomas while interrupting the *β*2M signalling pathway led to apoptosis of the tumour cells. Accordingly, the elevated expression of *β*2M may be associated with an increased resistance to apoptosis. As shown in Tables 1 and 2, an increase in the level of *β*2M during the progression of OCSCC is a sign of poor prognosis. The decreased level or total loss of immunoreactive staining for *β*2M may be due to the loss of the *β2M* locus, which has been shown to occur in early stages of lymph node-positive metastasising HNSCC lesions (Bockmuhl *et al*, 2002); mutations in the coding region of *β2M*, leading to a decrease or loss of *β*2M expression; or mutations in or methylation of the introns or promoter of *β2M*, leading to a decrease or total loss of expression (Feenstra *et al*, 1999a, 1999b; Koene *et al*, 2004).

Increased expression of *β*2M, as detected by immunohistochemical staining and/or shedding of the molecule into the urine and serum, has been observed in more advanced malignancies. This suggests that *β*2M modulates cellular proliferation as well as tumour cell migration and invasion. Thus, we generated two stable clonal cell lines of oral cancer cells, FaDu and SCC25, which overexpress *β*2M, and investigated whether *β*2M affected cell migration and invasion *in vitro*. Our results revealed a positive correlation between *β*2M expression and the migration and invasion of the *β*2M-overexprssing transfectants (Figure 3). In contrast, the inhibition of *β*2M expression by siRNA was sufficient to reduce cellular migration and invasion *in vitro* (Figure 4). These data are consistent with those showing increased immunoreactivity at more advanced stages of OCSCC (Figure 2 and Table 1), suggesting that a rise in the level of *β*2M facilitates tumour progression. Importantly, our immunohistochemical data showed that very weak intensity for *β*2M staining of almost of all normal oral mucosa was focally localised in the plasma membrane compared to that mainly found in the cytoplasm of tumour (∼90 to 92%) and the adjacent non-tumour tissues (∼80%). Although cytoplasmic staining of *β*2M has been demonstrated in some cases of human renal cell carcinoma (Nomura *et al*, 2006), here, we highlight the changes in *β*2M localisation from plasma membrane to cytoplasm between normal and tumour stages of OCSCC. As the association of *β*2M overexpression was significantly higher in those patients with OCSCC and lymph node metastasis (N+) than in those without lymph node metastasis (N-), *β*2M may promote metastasis in OCSCC. Our current findings agree with those from other reports showing that *β*2M is an effective growth-promoting factor in the growth and progression of renal cell carcinoma and prostate cancer (Huang *et al*, 2006; Nomura *et al*, 2006). Accordingly, these findings address the following clinical implications: (a) *β*2M must play a far-reaching function than just a housekeeping gene or the role on stabilisation and presentation of MHC class I molecule in cells; (b) *β*2M may act as an effective growth-promoting factor to facilitate tumour progression, invasion, and migration in OCSCC; and (c) increased synthesis and/or release of *β*2M by an elevated serum or urine *β*2M concentration may become one of important prognostic factor and survival predictors in OCSCC.

In conclusion, we found that *β*2M is aberrantly expressed in OCSCC relative to histologically adjacent non-tumour tissue. Moreover, *β*2M is an important factor for several clinicopathological variables in OCSCC, suggesting its potential as a biomarker of the disease. Furthermore, *β*2M overexpression facilitates the migration and invasion of oral cancer cells, which supports the finding that elevated levels of *β*2M are positively correlated with advanced OCSCC. Apart from the exploration of prognostic factors in OCSCC, our results present a potential target for immunotherapy.

## Figures and Tables

**Table 1:** Clinical profile and correlation between the clinicopathological features and intensity of *β*2M expression

		***β*2M staining**		
**Variables**	**No. of patients**	**Weak (−/+)**	**Strong (++/+++)**	***P*-value**
*Age (years)*				1
⩽59	202	57	145	
⩾60	54	15	39	
				
*Gender*				0.026
Male	239	63	176	
Female	17	9	8	
				
*Tumor stage*				<0.001^*^
T1, T2	94	69	25	
T3, T4	162	3	159	
				
*Nodal stage*				<0.001^*^
N(−)	153	67	86	
N(+)	103	5	98	
				
*TNM stage*				<0.001^*^
I, II	72	72	0	
III, IV	184	0	184	

^*^Statistically significant.

**Table 2:** Univariate analysis of various clinicopathological features and cumulative 5-year survival rates

**Variables**	**No. of patients**	**Cumulative 5-year survival rate (%)**	***P*-value**
*Age (years)*			
≦59	202	66.4	0.12
≧60	54	55.1	
			
*Gender*			0.14
Male	239	62.7	
Female	17	81.9	
			
*Tumor stage*			<0.001^*^
T1, T2	94	84.0	
T3, T4	162	52.3	
			
*Nodal stage*			<0.001^*^
N(−)	153	77.1	
N(+)	103	44.7	
			
*TNM stage*			
I, II	72	92.4	
III, IV	184	54.1	<0.001^*^
			
*β2M overexpression*			<0.001^*^
Weak	72	92.4	
Strong	184	54.1	

^*^Statistically significant.

**Table 3:** Multivariate survival analysis

**Variables**	**Hazard ratio (95% CI)**	***P*-value**
Tumor stage (T3, T4 *vs* T1, T2)	3.293 (1.877–5.778)	<0.001^*^
Nodal stage (N+ vs N−)	2.990 (1.937–4.616)	<0.001^*^

^*^Statistically significant.
