# Supplementary figures and images for: Overexpression of β2-microglobulin is associated with poor survival in patients with oral cavity squamous cell carcinoma and contributes to oral cancer cell migration and invasion
Source: Br J Cancer. 2008 Oct 7;99(9):1453–61. doi: 10.1038/sj.bjc.6604698 (PMC2579697; doi:10.1038/sj.bjc.6604698)

**Supplement 1**

**
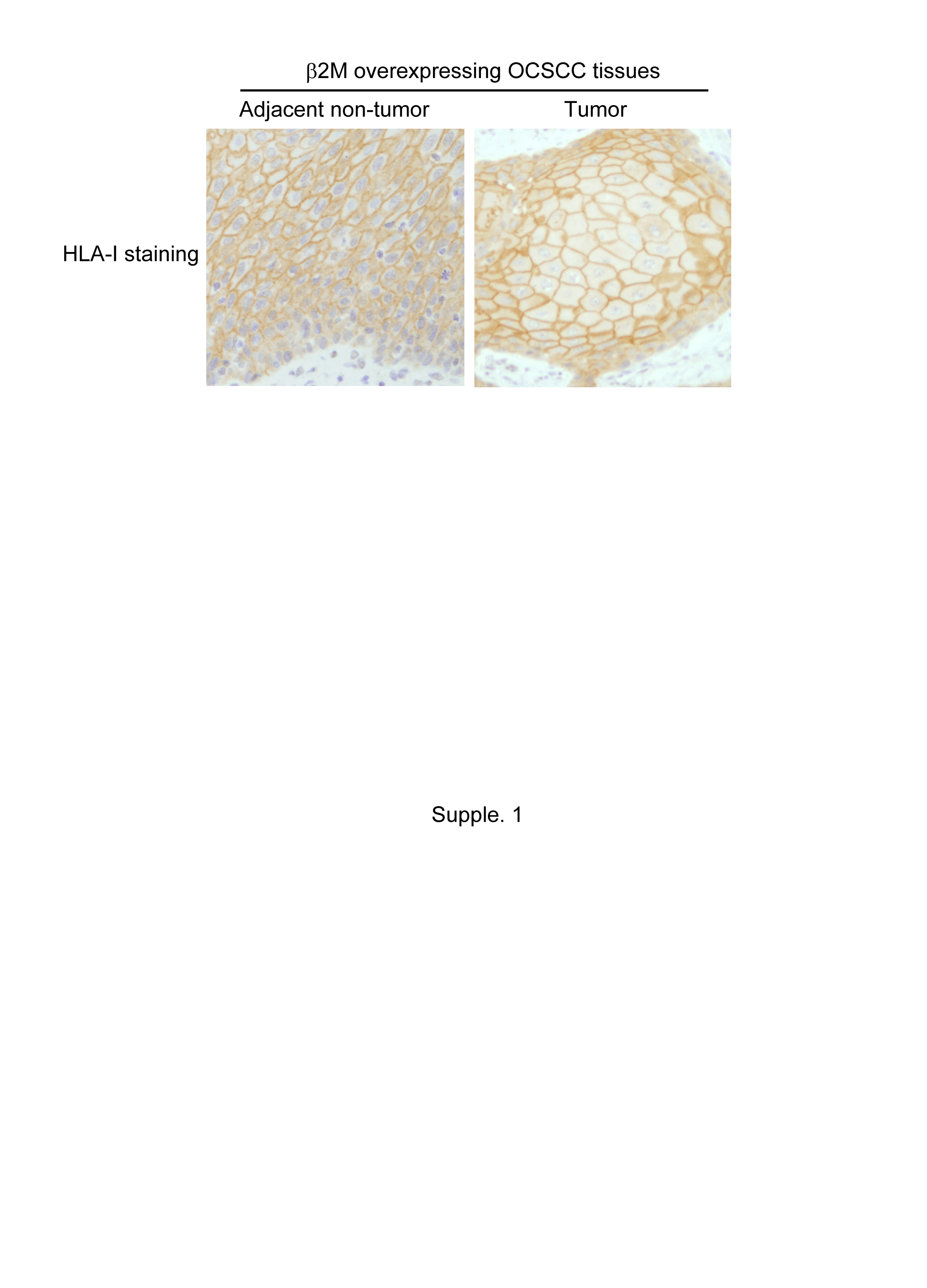
**

Supplement: Supplement 1 [file 6604698x1.doc]
