# Supplementary material for: Overexpression of β2-microglobulin is associated with poor survival in patients with oral cavity squamous cell carcinoma and contributes to oral cancer cell migration and invasion
Source: Br J Cancer. 2008 Oct 7;99(9):1453–61. doi: 10.1038/sj.bjc.6604698 (PMC2579697; doi:10.1038/sj.bjc.6604698)
Supplement: Supplement 1 Legend [file 6604698x2.doc]

**Figure Legends**

**Supplement 1.** Immunohistochemical staining of HLA-I protein expression in patients with OCSCC. Tissues slides of OCSCC patients were incubated with 1 mg/ml polyclonal antibody against HLA-I. The HLA-I staining intensity has no significantly different between adjacent-non tumor (left) and tumor tissues (right) in β2M-overexpressing specimens.
